# The impact of quality of work life, professional identity, and job burnout on presenteeism among family doctors: a cross-sectional study in China

**DOI:** 10.3389/fpubh.2026.1756164

**Published:** 2026-02-18

**Authors:** Wenxin Yu, Min Liu, Zihang Gao, Zijian Qi, Chunxia Miao, Wenjun Yan, Wei Wang, Xiuyin Gao, Qingzhi Wang

**Affiliations:** 1 Department of Community and Health Education, School of Public Health, Xuzhou Medical University, Xuzhou, China; 2 Department of Health Management, School of Management, Xuzhou Medical University, Xuzhou, China; 3 Center for Medical Statistics and Data Analysis, Xuzhou Medical University, Xuzhou, China

**Keywords:** family doctors, job burnout, presenteeism, professional identity, quality of work life

## Abstract

**Background:**

To foster high-quality healthcare, family doctors plays a crucial role in China's primary healthcare system. Facing dual pressures in medical care and public health, family doctors are vulnerable group to presenteeism. This study aims to explore their quality of work life, professional identity, job burnout and the presenteeism, and analyzes the factors affecting the presenteeism.

**Methods:**

A cross-sectional survey was conducted with 731 family doctors from May 2021 to April 2022. Pearson correlation analysis assessed the correlation between quality of work life, professional identity, job burnout and presenteeism, and multiple linear regression analysis determined the influencing factors of presenteeism.

**Results:**

The Work-Related Quality of Life and Professional Identity displayed negative correlations with the presenteeism (*p* < 0.01), and the job burnout showed positive correlations with the presenteeism (*p* < 0.01). Hierarchical linear regression revealed significant impacts of work stress (β = −0.144, *p* = 0.033), tendency to professional behavior (β = −0.239, *p* < 0.001), professional values (β = −0.115, *p* = 0.042), sense of professional belonging (β = −0.198, *p* = 0.004), emotional exhaustion (β = 0.168, *p* = 0.009), depersonalization (β = 0.183, *p* = 0.006), and professional efficacy (β = 0.148, *p* = 0.033) on presenteeism.

**Conclusions:**

The study indicates that the higher the quality of work life, the higher the level of professional identity and the lower the level of job burnout, the lower will be the level of presenteeism among family doctors. Addressing these elements through targeted interventions, such as dynamic talent allocation, enhancing professional identity, and mitigating burnout, could reduce presenteeism.

## 1 Introduction

After decades of evolution, the family doctor system has matured in countries such as the United Kingdom (1), the United States (2), Australia (3) and Canada (4). In contrast, the family doctor system is still undergoing continual development. Since the healthcare reforms initiated in 2009, China has entered a new phase in its medical system, actively encouraging residents to register with family doctor teams, who have become the primary ‘gatekeepers' of healthcare for the population (5). Subsequently, in 2016, seven departments, led by the Medical Reform Office of the State Council, jointly formulated the Guiding Opinions on Promoting Family Doctor Signing Service (6), which facilitates the downward shift of the center of gravity of medical and healthcare work and the redistribution of resources through the contracted services of family doctors, and guides the public to seek medical treatment in an orderly manner. This initiative aims to further address the imbalance in the development of primary healthcare resources and tackle the challenges posed by the aging population, such as the difficulties in accessing and affording medical care (7). As the Healthy China initiative gains momentum, the demand for family doctors is rising, as they play a crucial role in healthcare and public health services. Family doctors are essential in guiding patients regarding the necessity and appropriateness of hospital visits (8).

Against this backdrop, presenteeism has attracted widespread attention within the field of healthcare management. Presenteeism refers to the phenomenon in which individuals, despite feeling unwell and experiencing health issues, continue to attend work (9). It is estimated that productivity losses due to presenteeism are more than four times greater than those due to presenteeism (10). Research indicates that the incidence of presenteeism is higher in the healthcare sector compared to other industries (11). A previous study shows that presenteeism is linked to negative outcomes for patients, nurses, and healthcare organizations (12). Studies have demonstrated that emergency staff exhibit high rates of presenteeism (13). Additionally, research has identified a prevalent trend of presenteeism among doctors in Chinese hospitals, which is notably associated with increased instances of anxiety or depression (14). However, a systematic review of existing literature reveals a notable gap in current research: although presenteeism in the healthcare sector has been partially explored, targeted and in-depth studies specifically addressing presenteeism among family doctors remain scarce.

As ‘gatekeepers' of primary healthcare, family doctors face unique work pressures characterized by heavy caseloads, complex service requirements, and high demands for emotional labor. These factors negatively impact their professional performance (15) and differ from the experiences of hospital doctors and emergency department staff. Neglecting research into presenteeism among this critical group not only hinders a comprehensive understanding of presenteeism across the healthcare sector but also impedes the development of targeted interventions to enhance family doctors' job satisfaction and service quality. Given their pivotal role in the sustained development of primary healthcare systems (16), investigating the current state of presenteeism among family doctors and its influencing factors holds significant practical importance for optimizing primary healthcare services, promoting the healthy development of the family doctor system, and advancing the ‘Healthy China' strategy.

To address the aforementioned research gap, this study aims to investigate the prevalence of presenteeism among family doctors in China and its key influencing factors through a large cross-sectional survey. This study will employ a questionnaire survey method, utilizing standardized presenteeism measurement tools and multidimensional influencing factor scales, to conduct a sample survey of family doctors. Through statistical analysis, multiple factors significantly associated with presenteeism have been identified, thereby providing an empirical basis for conducting further research to explore its causes, consequences, and potential interventions.

## 2 Materials and methods

### 2.1 Participants

A representative sampling method was utilized to select community health service centers in Xuzhou City, Jiangsu province, that have implemented family doctor services programs. The selected districts included Gulou, Quanshan, Tongshan, Yunlong, as well as Pei County, Feng County, and Pizhou County, comprising a total of 46 centers. The study involved all family team members across these centers, numbering 800 participants. The criteria for participation were as follows: inclusion was limited to medical practitioners, nurses and public health physicians who were either internally or externally hired and actively engaged in family doctor services. The exclusion criteria were: (1) General practitioners, specialists, public health physicians and nurses who are not involved in the family doctor services. (2) Non-medical personnel associated with the family doctor teams.

### 2.2 Data collection

The study was carried out over a period of one year, from May 2021 to April 2022, with the assistance of investigators who had undergone specialized training. The data collection involved face-to-face interview, in which the investigators provided a detailed explanation of the survey's objectives and procedures to the participants. They also guided the participants through the process of completing the questionnaire, emphasizing the necessary steps and any precautions to be taken. Once the participants had comprehended and consented to the survey, they independently filled out the questionnaires. Subsequently, the investigators reviewed and verified the completed questionnaires to ensure their accuracy and to maintain the integrity of the data collected. This study was approved by the Ethics Committee of Xuzhou Medical University and it was conducted in accordance with the Declaration of Helsinki. All participants understood the research purpose, process, potential risks, and benefits, and they agreed to participate in this study by providing written informed consent.

### 2.3 Instruments

In conjunction with the purpose of the study, the questionnaire was designed through literature review, expert consultation and team discussion. The main components of the questionnaire consisted of a general information questionnaire for family doctors and related instrumental scales, including the Work-Related Quality of Life (WRQoL), the Professional Identity Scale (PIS), Maslach Burnout Inventory-General Survery (MBI-GS), and the Stanford Presenteeism Scale-6 (SPS-6).

#### 2.3.1 General information questionnaire

This includes the family doctor's sex, age, education, years of service, marital status, title, monthly income, and type of employment.

#### 2.3.2 Work-Related Quality of Life (WRQoL)

The WRQoL designed by Van Laar (17) at the University of Portsmouth, UK, can be used to assess the quality of work-life of healthcare professionals. This scale assesses multiple facets of work life that contribute to overall wellbeing and satisfaction within a professional setting. In this study, five key dimensions from the WRQoL were selected as primary indicators to evaluate family doctors' quality of work life: job satisfaction, work environment, family-work relationship, work stress, and work control. Participants responded to each item on a five-point Likert scale ranging from 1 (strongly disagree) to 5 (strongly agree), allowing for a nuanced view of attitudes toward various aspects of work life. Notably, items under the work stress dimension were reverse-scored to account for the adverse effects of stress on work-life quality. Thus, higher scores across these dimensions reflect greater levels of quality in work life, indicating a healthier and more supportive professional environment. In this study, the WRQoL demonstrated good internal consistency, with an overall Cronbach's α of 0.751.

#### 2.3.3 Professional Identity Scale (PIS)

The scale used in this study integrates key contents of professional identity assessment developed by Hao Yufang (18) and Wei Shuhua (19) to comprehensively measure professional identity. It encompasses four dimensions: professional self-awareness, tendency to professional behavior, professional values, and sense of professional belonging, which were used as observational variables to respond to professional identity. The questionnaire consisted of 17 items, and each item were rated on a five-point Likert scale from 1 (strongly disagree) to 5 (strongly agree). In this study, the scale achieved a Cronbach's α of 0.673, indicating acceptable internal consistency for measuring professional identity.

#### 2.3.4 Maslach Burnout Inventory-General Survey (MBI-GS)

The Maslach Burnout Inventory-General Survey (MBI-GS) was originally developed by Maslach (20), and the Chinese version of the Maslach Burnout Scale was validated by scholars as early as 2003 (21) and has been widely used in China. In this study, the questionnaire was adapted to make it more suitable for assessing burnout levels in family doctors. The MBI-GS is a well-validated instrument designed to measure burnout across three primary dimensions: emotional exhaustion, depersonalization, and professional efficacy. This scale consists of 15 items that capture a range of experiences related to professional stress and burnout. Responses were recorded on a seven-point Likert scale, with options ranging from ‘never' (0) to ‘every day' (6). Higher scores on this scale indicate a more severe degree of burnout across the dimensions, signaling greater emotional exhaustion, depersonalization, and lower professional efficacy. In the present study, the MBI-GS demonstrated strong internal consistency, achieving a Cronbach's α of 0.851, which underscores the reliability of this instrument in capturing burnout among the surveyed population.

#### 2.3.5 Stanford Presenteeism Scale-6 (SPS-6)

The Stanford Presenteeism Scale-6 (SPS-6) is a widely used tool developed to measure presenteeism, initially developed by Koopman (22) in 2002. The SPS-6 was later adapted and translated into Chinese, where it has demonstrated robust reliability and validity within various professional populations in China, making it a useful tool for understanding presenteeism in this context (23). The scale comprises six items and participants respond to each item using a five-point Likert scale, ranging from 1 (strongly disagree) to 5 (strongly agree). The total score on the SPS-6 can range from 6 to 30, with higher scores indicating a greater impact of health-related challenges on work productivity. In this study, the SPS-6 exhibited an acceptable level of internal consistency, with a Cronbach's α of 0.731, supporting its reliability in capturing presenteeism-related productivity loss.

### 2.4 Statistical methods

The basic demographic characteristics of the participants were described using frequencies and percentages [n (%)]. The scores of presenteeism, work-related quality of life (WRQoL), professional identity, and professional burnout were summarized as means ± standard deviations (M ± SD). Differences in presenteeism across individual characteristics were analyzed using independent samples *t*-tests or one-way ANOVA, as appropriate. Pearson correlation analysis was used to assess the relationships between presenteeism, WRQoL, professional identity, and professional burnout. To identify the factors influencing presenteeism, a hierarchical regression analysis was performed: Model 1: the explanatory variables included the dimensions of WRQoL, while participants' sociodemographic characteristics were included as covariates. Model 2: the dimensions of professional identity were added to the variables in Model 1. Model 3: the dimensions of professional burnout were further added to the variables in Model 2. Statistical analysis was conducted using SPSS 23.0 software, with statistical significance set at *p* < 0.05.

## 3 Results

### 3.1 Basic information on family doctors

A total of 800 questionnaires were distributed and 731 valid questionnaires were collected, with a valid response rate of 91. 38 %. As shown in Table 1, of the 731 family doctors, 70.9%were women (12.37 ± 4.55); 31.2% were aged 31–40 (12.27 ± 4.55); 55.7% had an undergraduate degree (12.72 ± 4.35); 81.7% were married (12.45 ± 4.46); and 41.3% had a primary title (11.79 ± 4.44); monthly incomes of RMB 2,001–4,000 (11.59 ± 4.52) and RMB 4,001–6,000 (13.33 ± 4.11) accounted for 47.1 and 30.6%, respectively; 55.4% were long-term employed (11.47 ± 4.60).

**Table 1:** Basic information on family doctors.

**Characteristics**	**n (%)**	**Presenteeism**	***F/t***	***p***
**Gender**				
Male	213 (29.1)	12.41 ± 4.49	0.128	0.898
Female	518 (70.9)	12.37 ± 4.55		
**Age (years)**				
≤ 30	198 (27.1)	11.47 ± 4.58	5.14	0.002
31–40	228 (31.2)	12.27 ± 4.55		
41–50	227 (31.0)	13.13 ± 4.12		
≥51	78 (10.7)	12.87 ± 4.90		
**Education level**				
High school or below	58 (7.9)	12.81 ± 3.70	3.128	0.025
Junior college	258 (35.3)	11.72 ± 4.88		
Undergraduates	407 (55.7)	12.72 ± 4.35		
Postgraduates	8 (1.1)	14.00 ± 5.10		
**Job tenure (years)**				
1–5	176 (24.1)	11.47 ± 4.48	5.277	< 0.001
6–10	145 (19.8)	12.66 ± 4.87		
11–20	178 (24.4)	11.84 ± 3.96		
21–30	172 (23.5)	13.39 ± 4.32		
31 and above	60 (8.2)	13.17 ± 5.29		
**Marital status**				
Unmarried	120 (16.4)	11.94 ± 4.75	0.879	0.416
Married	597 (81.7)	12.45 ± 4.46		
Divorce or widowhood	14 (1.9)	13.21 ± 5.38		
**Professional title**				
None	63 (8.6)	11.05 ± 4.22	6.500	< 0.001
Primary	302 (41.3)	11.79 ± 4.44		
Medium	255 (34.9)	12.87 ± 4.53		
Senior	92 (12.6)	13.28 ± 4.49		
High	19 (2.6)	15.32 ± 4.46		
**Monthly income**				
≤ 2,000	87 (11.9)	12.29 ± 4.85	8.129	< 0.001
2,001–4,000	344 (47.1)	11.59 ± 4.52		
4,001–6,000	224 (30.6)	13.33 ± 4.11		
≥6,000	76 (10.4)	13.30 ± 4.72		
**Type of employment**				
Temporary employee	326 (44.6)	13.52 ± 4.18	6.295	< 0.001
Long-term employee	405 (55.4)	11.47 ± 4.60		

### 3.2 Correlation analysis of quality of work life, professional identity, job burnout, and presenteeism

There was a positive correlation between the total score of WRQoL and its five dimensions and the total score of PIS and its four dimensions (*p* < 0.01); Except for the correlation between job control and emotional exhaustion, which was not statistically significant, there was a negative correlation between the total score of WRQoL and its five dimensions, the total score of PIS and its five dimensions, and the total score of MBI-GS and its three dimensions (*p* < 0.01); Except for the dimension of job control, the total score of WRQoL and all other dimensions, the total score of PIS and its five dimensions were negatively correlated with the presenteeism score, and the total score of MBI-GS and its three dimensions were positively correlated with the total score of SPS-6 (*p* < 0.01) as shown in Table 2.

**Table 2:** Correlation analysis of quality of work life, professional identity, job burnout, and presenteeism.

**Items**	**(M ±SD)**	**Presenteeism**	
		***R***	***p***
**Quality of work life**			
Work satisfaction	19.08 ± 3.30	−0.234^**^	0.008
Work environment	18.77 ± 3.46	−0.364^**^	< 0.001
Work-family relationship	10.51 ± 2.43	−0.367^**^	< 0.001
Work stress	3.27 ± 1.71	−0.404^**^	< 0.001
Work control	4.98 ± 1.64	0.007	0.862
**Professional identity**			
Professional self-awareness	22.72 ± 5.05	−0.295^**^	0.006
Tendency to professional behavior	22.04 ± 3.50	−0.526^**^	< 0.001
Professional values	12.65 ± 2.07	−0.471^**^	< 0.001
Sense of professional belonging	13.53 ± 2.15	−0.426^**^	< 0.001
**Job burnout**			
Emotional exhaustion	9.18 ± 5.84	0.537^**^	< 0.001
Depersonalization	6.23 ± 5.37	0.606^**^	< 0.001
Professional efficacy	11.69 ± 8.41	0.447^**^	< 0.001

^**^denotes p < 0.01.

### 3.3 Multiple linear regression analysis of presenteeism

Table 3 shows the factors influencing presenteeism derived from the multiple linear regression analysis. In Model 1, work environment (β = −0.154, *p* = 0.027), work-family relationship (β = −0.200, *p* = 0.004), and work stress scores (β = −0.283, *p* < 0.001) had a negative effect on presenteeism when we included the scores of the WRQoL dimensions and basic information about the family doctors in the regression analysis. Model 2 expands the analysis by adding the PIS dimension score to Model 1, and work-family relationships are no longer significant for presenteeism. Instead, tendency to professional behavior (β = −0.284, *p* < 0.001), professional values (β = −0.132, *p* = 0.038), and sense of professional belonging (β = −0.256, *p* < 0.001) have a negative effect on presenteeism. Model 3 continues to expand the analysis by adding the MBI-GS dimension scores to Model 2. The results show that work environment is also no longer significant on presenteeism. Instead, emotional exhaustion (β = 0.168, *p* = 0.009), depersonalization (β = 0.183, *p* = 0.006), and professional efficacy (β = 0.148, *p* = 0.033) had a positive effect on presenteeism. Marital status and type of employment were statistically significant in all model.

**Table 3:** Multiple linear regression analysis of presenteeism.

**Variables**	**Model 1**		**Model 2**		**Model 3**	
	**β**	***P***	**β**	***p***	**β**	***p***
Work satisfaction	−0.078	0.273	0.028	0.649	0.035	0.427
Work environment	−0.154	0.027	−0.107	0.036	−0.020	0.716
Work-family relationship	−0.200	0.004	−0.075	0.276	−0.048	0.382
Work stress	−0.283	< 0.001	−0.218	0.001	−0.144	0.033
Professional self-awareness			−0.068	0.314	−0.016	0.674
Tendency to professional behavior			−0.284	< 0.001	−0.239	< 0.001
Professional values			−0.132	0.038	−0.115	0.042
Sense of professional belonging			−0.256	< 0.001	−0.198	0.004
Emotional exhaustion					0.168	0.009
Depersonalization					0.183	0.006
Professional efficacy					0.148	0.033
*R^2^*	0.298		0.523		0.617	
Adjusted *R^2^*	0.285		0.511		0.605	

Control variables include gender, age, education, years of service, marital status, title, position, monthly income, and presence of establishment.

All the variance inflation factors (VIF) of the variables included in the regression analysis are less than 5.

## 4 Discussion

The study provides valuable insights into the work-related challenges encountered by family doctors in China. Through a comprehensive cross-sectional study, we found that a higher quality of work life among family doctors is associated with a higher level of professional identity and a lower level of job burnout, resulting in reduced productivity losses due to presenteeism. To the best of our knowledge, this is the first study in China to investigate the phenomenon of presenteeism among family doctors and its influencing factors from multiple dimensions. Our research aims to provide scientific evidence to enhance the work motivation and medical quality of family doctors.

Stress is an inevitable consequence of the development of modern society that can disrupt or threaten an individual's mental state, accompanied by a range of emotional and physical tensions and discomforts. This study demonstrates that job stress has a significant impact on presenteeism, consistent with findings from several studies (24, 25). Work stress has been recognized as a critical factor closely related to presenteeism (26, 27). According to a study by Liangwen Ning (28), work stress may compel employees to continue working despite feeling unwell, which may lead to increased presenteeism and reduced commitment to the primary healthcare organization, ultimately increasing the intent to resign. The physical and mental health of family doctors is particularly vulnerable to workplace stressors, thereby increasing the risk of presenteeism. These stressors originate from multiple dimensions, including excessive clinical workloads, substantial administrative burdens, and limited societal recognition (7). Thus, mitigating work-related stress among general practitioners calls for systematic, multilevel interventions. Administrators should enhance talent cultivation, improve social security, and provide greater support to family doctors (29) while fostering positive public perception to create a calm and relaxed work environment, mitigating the negative effects of job stress (30). Furthermore, managers should also consider other unmeasured factors, including management style, professional development of family doctors and salary and benefits. This will effectively reduce the stress of the family doctors' work and minimize the incidence of presenteeism.

Professional identity was negatively correlated with presenteeism, indicating that higher professional identity among family doctors is associated with a greater sense of value and belonging to their profession, as well as an increased recognition of their role, resulting in lower levels of presenteeism. Model 3 illustrates that tendency to professional behavior, professional values and sense of professional belonging all have a negative effect on presenteeism. Research on professional identity suggests that it is a central component of identity formation, reflecting one's values, beliefs, and commitment to work, which in turn helps individuals excel in their careers (31). Social identity theory posits that identity can significantly influence emotions, attitudes and behaviors (32). Family doctors with high professional identity typically develop a clearer perception of the social value of their work, which in turn fosters a stronger sense of responsibility and greater emotional resilience. As a result, they are more capable of maintaining effective work performance under pressure. This finding implies that relying exclusively on external incentives may not be sufficient to sustain improved work performance. It is therefore essential to enhance family doctors' intrinsic professional identity through measures such as vocational training, mentorship programs, and the cultivation of a supportive team culture. Therefore, it is recommended that administrators provide enhanced guidance on career planning for family doctors and help them establish sound professional values, fostering positive recognition and respect for their work, which may ultimately improve professional identity and reduce presenteeism.

This study indicates a significant positive correlation between presenteeism and job burnout among family doctors. Model 3 indicates that after introducing control variables and factors related to work life quality and professional identity, independent correlations among the three dimensions of job burnout and presenteeism persist. This suggests a stable correlation between presenteeism and the dimensions of emotional exhaustion, depersonalization, and professional efficacy, unaffected by demographic factors, work nature, personal ideology, etc. These findings align with the results of Pei Pei's study (33) on presenteeism and job burnout among Chinese doctors. It also aligns with the principles of Conservation of Resources theory, which holds that sustained depletion of emotional and cognitive resources reduces an individual's effective work capacity, thereby increasing the likelihood of presenteeism, a state characterized by physical presence accompanied by diminished cognitive and emotional engagement (34). Presenteeism serves as an effective indicator of an employee's actual work output over time. Individuals experiencing high levels of job burnout are prone to losing enthusiasm for their work, which leads to decreased job performance (35); conversely, poor job performance may result in increased presenteeism. Therefore, intervention measures must address both organizational and individual levels. At the organizational level, workload should be distributed reasonably, with performance feedback and career support provided. At the individual level, psychological intervention programs such as stress management may be introduced to help family doctors enhance their emotional regulation capabilities.

Our study investigated presenteeism among family doctors and the relationship between the three dimensions of quality of work life, professional identity, and job burnout. To the best of our knowledge, few domestic and international scholars have studied this topic, and most have focused on invisible presenteeism among clinicians, nurses, and general practitioners, examining the relationship from only one or two dimensions. In contrast, our study is one of the pioneers in exploring this relationship comprehensively. Thus, our findings contribute to the limited evidence available on this topic. However, it is important to acknowledge that our study has several limitations. First, as a cross-sectional study, it could not establish causal associations between the influencing factors and presenteeism; therefore, future longitudinal studies are necessary to validate these associations. Second, the reliance on self-reported data from family doctors may introduce a Hawthorne effect, potentially underestimating presenteeism; thus, future observational studies are warranted to accurately assess the extent of presenteeism among family doctors. Third, the scope of this study is confined to Xuzhou City, which limits the generalizability and applicability of the findings to other settings; Furthermore, due to the choice of measurement tools, relevant organizational and contextual variables such as workplace atmosphere, management style, and institutional policies were not included in the analysis. Therefore, larger-scale surveys across multiple provinces will be conducted in the future to address this limitation.

## 5 Conclusion

The study provides valuable insights into the presenteeism faced by family doctors in China. The findings indicate quality of work life, professional identity have a significant negative impact, implying that support in these areas could potentially reduce presenteeism. On the other hand, emotional exhaustion, depersonalization, and professional efficacy had a positive impact, suggesting that interventions targeting these aspects of job burnout could be beneficial. The study underscores the need for targeted interventions to enhance work life quality, foster professional identity, and mitigate job burnout to reduce presenteeism among family doctors. This is crucial for maintaining a healthy and productive primary healthcare workforce in China.

## Data Availability

The original contributions presented in the study are included in the article/supplementary material, further inquiries can be directed to the corresponding authors.
